# Effects of psychological interventions on high sports performance: A systematic review

**DOI:** 10.3389/fpsyg.2022.1068376

**Published:** 2022-12-20

**Authors:** Mario Reyes-Bossio, Santiago Corcuera-Bustamante, Giancarlo Veliz-Salinas, Marcelo Villas Boas Junior, Mariel Delgado-Campusano, Paul Brocca-Alvarado, Tomás Caycho-Rodríguez, Leslie Casas-Apayco, Veronica Tutte-Vallarino, Carlos Carbajal-León, Regina Brandão

**Affiliations:** ^1^Facultad de Psicología, Universidad Peruana de Ciencias Aplicadas, Lima, Peru; ^2^Universidade São Judas Tadeu, São Paulo, Brazil; ^3^Universidad Científica del Sur, Lima, Peru; ^4^Facultad de Ciencias de la Salud, Universidad Privada del Norte, Lima, Peru; ^5^Departamento de Bienestar y Salud, Universidad Católica del Uruguay, Montevideo, Uruguay; ^6^South American Center for Education and Research in Public Health, Universidad Norbert Wiener, Lima, Peru

**Keywords:** sports psychology, intervention programs, athletes, high performance, psychological skills

## Abstract

**Introduction:**

Intervention programs in sports psychology aid to modify the thoughts and behaviors of athletes in order to improve their performance in sports settings. For high-performance athletes, these interventions are very relevant, given that they constantly face pressure towards obtaining sporting achievements.

**Methods:**

This systematic review aims to analyze the scientific articles between 2010 and 2020 that evaluated the effect of psychological interventions on high-performance athletes. In the search procedure, nine studies were selected, the most studied variables were psychological skills, psychological flexibility, and stress.

**Results:**

The 44% of the interventions were designed by the research authors themselves, while the remaining 56% were replicated programs, which already had scientific evidence.

**Discussion:**

Psychological interventions have a positive impact on sports performance. This review allows sports institutions and professionals to have more knowledge and resources at their disposal to implement these types of programs in their sports planning.

## Introduction

The psychology of sports and physical exercise is a scientific discipline that focuses on the study of people behaviors in the context of sports and physical activities (Gill, 2000). In addition, it includes the application of psychological theories to understand and optimize the performance, mental processes, and wellbeing of these people (Moran and Toner, 2017). In particular, the discoveries linking mental aspects and athletic performance were originated almost simultaneously with those of psychology (Kornspan, 2011; Kremer et al., 2012). Nevertheless, since decades ago this discipline has been consolidated and has experienced a considerable growth, accompanied by greater activity and relevance in scientific production (Weinberg and Gould, 2010; Berengüi and López-Walle, 2018). This increase has allowed sports agents to have at their disposal multiple scientifically validated knowledge and resources to solve daily practical questions related to sports performance (Moran and Toner, 2017). However, one of the great challenges for sports psychology is to continue updating the knowledge obtained and to be in constant search of new subfields that answer its main questions (Cantón, 2016). A convenient way to do this is through the development of intervention programs with a specific methodology that includes evaluation instruments, techniques, and strategies, both direct and indirect (Cantón, 2010).

An intervention program can be defined as an action or process that affects the functioning and/or performance of a person through changes in their thinking and behavior (American Psychological Associaton, 2002). In the specific case of sports psychology and physical exercise, this is achieved through psychological factors related to the sports context.

Sports psychological interventions have proven to be important over the years given the positive impact that they have on wellbeing and the optimization of sports performance (Greenspan and Feltz, 1989; Weinberg and Comar, 1994; Martin et al., 2005; Brown and Fletcher, 2017). In addition, the training and/or learning of strategies and techniques acquired in these interventions, allow the development of psychological skills such as concentration, activation level, motivation and other cognitive skills required for the most demanding sports scenarios (Craig, 2011; Olmedilla et al., 2011, 2018; Escolano et al., 2014; Olmedilla and Domínguez-Igual, 2016; Larkin et al., 2018; McCromick et al., 2018).

Previous literature on other systematic reviews have emphasized the impact of psychological variables on sports performance. For instance, Brown and Fletcher (2017) carried out a meta-analysis that aimed to synthesize all the studies, without a defined range of years, that evaluated the psychological, social, and psychosocial interventions with athletes and the relationship they had with their athletic performance. On the other hand, Ursino and Barrios (2019) described how sports performance associated with psychological variables has been studied, although it focused on all empirical studies in general. These scientific evidences have become known thanks to the fact that these and other sports science professionals have undergone very rigorous research processes, in which they conceptualized, developed, intervened, and evaluated the impact of certain psychological skills on athletes' performance. Therefore, to continue contributing to the spread and growth of this discipline, the purpose is to carry out a systematic review of the most recent studies that have evaluated the effects of psychological interventions on the performance of professional athletes in sports settings, high competition, or high-performance athletes.

The Instituto Peruano del Deporte (2019) groups these people into the category called high-level qualified athlete, which means that they represent the country in official international events and obtain results in them. However, for the purposes of this research, the high-performance athlete has been defined as an athlete who can also represent a professional club through competitive tournaments, regardless of their category.

It should be noted that other recent systematic investigations have been carried out, which emphasized the impact of psychological variables on sports performance. On the one hand, Brown and Fletcher (2017) carried out a meta-analysis that aimed to synthesize all the studies, without a defined range of years, that evaluated the psychological, social, and psychosocial interventions with athletes and the relationship they had with their athletic performance. On the other hand, Ursino and Barrios (2019) described how sports performance associated with psychological variables has been studied, although it focused on all empirical studies in general.

In this sense, the purpose of this review was to synthesize and analyze the scientific studies between the period 2010–2020 that have evaluated the effects of psychological interventions on the performance of professional athletes who perform in highly competitive settings.

## Methods

### Protocol

This systematic review followed the guidelines of the methodology proposed in the PRISMA statement (Preferred Reporting Items for Systematic reviews and Meta-Analyses) (Moher et al., 2009).

### Search strategy

The studies were identified in electronic databases with scientific relevance and good contribution to the spread of research in the psychology of sports and physical exercise. The search was carried out in WEB OF SCIENCE, SCOPUS, SCIELO and PSICODOC using the following keywords: “sport psychology” AND “intervention program,” including the Boolean operators “AND” between these keywords and the concept “high performance” and excluding through “NOT” recreational sport, physical exercise, case and/or single case studies and interventions that delve into physiological aspects. We considered the use of keywords in English because the results were more numerous with this search, finding research in other languages as well, such as Spanish and Portuguese. In relation to time limiters we selected a 10-year period, between 2010 and 2020. Finally, we chose full-text publications, leaving aside abstracts, as specified in the PRISMA system.

### Inclusion criteria

The articles were selected with the following inclusion criteria: (1) intervention programs that contemplate a pre-test, intervention, and post-test in their analysis; (2) investigations that evaluate the effect on psychological variables related to the optimization of sports performance; (3) sample in high-performance athletes who participate in professional categories in sports and indistinct ages; (4) the years between publication of the studies is from 2010 to 2020; and (5) the evaluation of interventions can be both quantitative and qualitative. It should be noted that the language or origin country did not compromise the inclusion of any study in the subsequent analyzes because greater cultural diversity was search among the articles searched.

### Study selection

**Figure 1:**
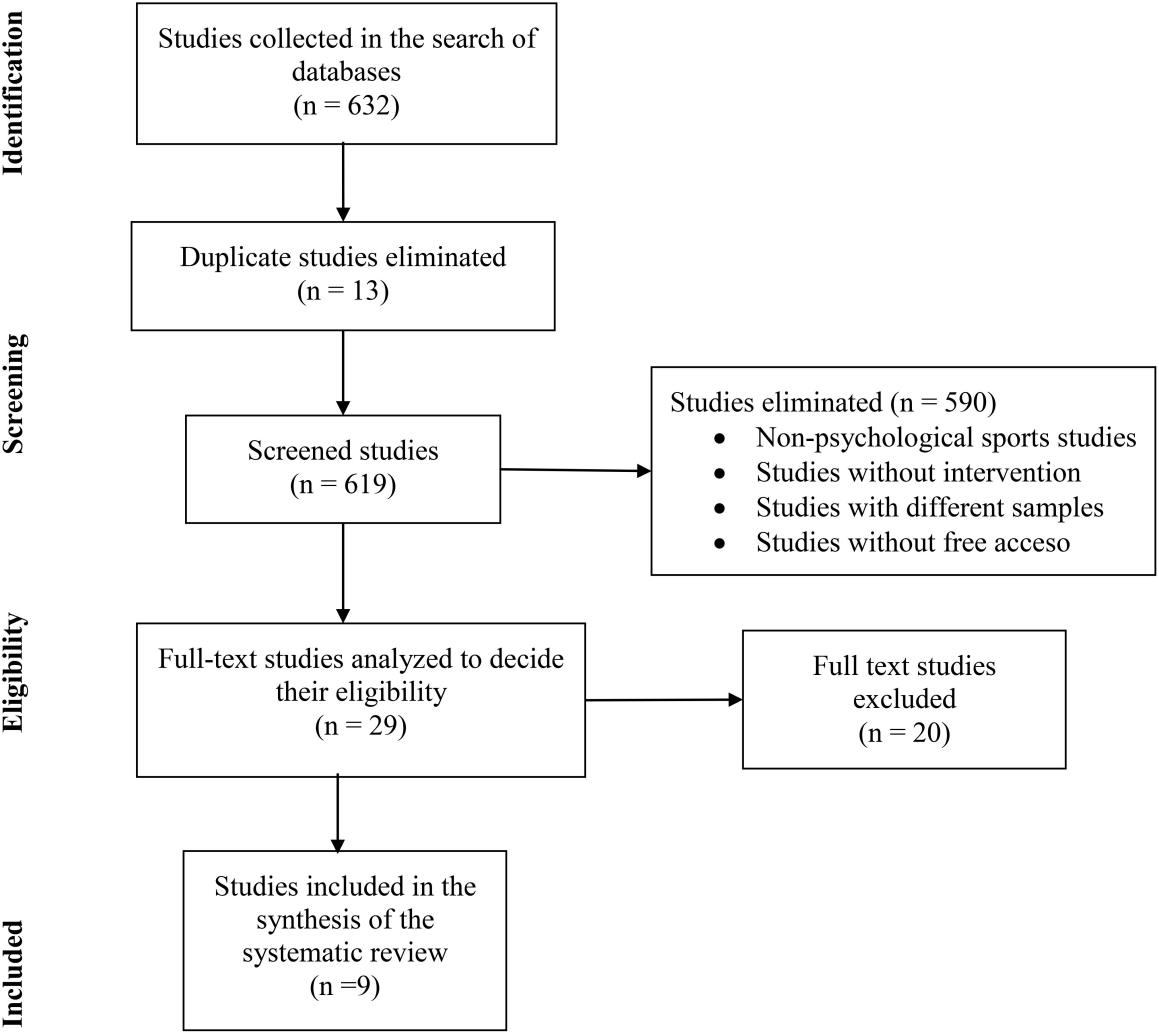
PRISMA flow chart for study selection.

The details of the research compilation strategy and its different phases are shown in the PRISMA flow diagram (Figure 1), while the references and the most relevant data were also rigorously reviewed through databases at Microsoft Excel. In the first phase, 632 articles were identified, using the keywords described above and in the selected electronic databases. Of this total, 13 duplicate articles were detected, so that in the second phase they were erased. In this same study, other 590 investigations were also excluded because, through the reading of titles and abstracts, they did not belong to the discipline of sports psychology, they did not present any type of intervention (correlational, comparative designs, reviews, etc.), their sample was not with athletes (coaches, parents, referees, etc.), and finally, others who did not have free access. In this way, 603 investigations were excluded, which, subtracted from the 632 found, result in 29 research that were maintained in the present systematic review.

In the third phase, the authors read the full text of the 29 selected articles to analyze and decide their eligibility. From this, 16 works were eliminated because they did not meet the established criteria. In most cases, these were excluded because the participant athletes were not considered as high-performance athletes (e.g., university, school, training academy athletes, etc.). In addition, other articles only reported or described an intervention, without evaluating it (pre-test and post-test). Also, some works selected nutritional, physical, or biomechanical variables to evaluate the impact on sports performance. Finally, four of the 13 remaining articles were eliminated to obtain greater uniformity in the discussion of results. Therefore, two studies with a qualitative design and two with single case studies were deleted. As a result of this, in the fifth phase, nine articles were included in the systematic review for further analyzes shown in the results (Reyes-Bossio et al., 2012; García-Naveira, 2016; Sallen et al., 2018; Campo et al., 2019; Carraça et al., 2019; Holguín-Ramírez et al., 2020; Lundgren et al., 2020; Tutte Vallarino et al., 2020; Vidarte et al., 2020).

### Quality assessment

The quality assessment was carried out based on the STROBE statement for observational methodological designs (Von et al., 2014). It comprehends a checklist of items that should be included in the case study and control reports. This format is made up of 31 variables assigned to sections (a) title and summary, (b) introduction, (c) method, (d) results, (e) discussion and (f) financing information, answered dichotomously among themselves or does not meet the quality attribute (Table 1).

**Table 1:** STROBE statement—checklist of items that should be included in reports of case-control studies.

**Section**	**Items no**	**Recommendations**	**Investigations**								
			**1**	**2**	**3**	**4**	**5**	**6**	**7**	**8**	**9**
Title and abstract	1	(*a*) Indicate the study's design with a commonly used term in the title or the abstract	x	x	x				x	x	x
		(*b*) Provide in the abstract an informative and balanced summary of what was done and what was found	x	x	x	x	x	x	x	x	x
Introduction											
Background/rationale	2	Explain the scientific background and rationale for the investigation being reported	x	x	x	x	x	x	x	x	x
Objectives	3	State specific objectives, including any prespecified hypotheses	x	x		x	x	x	x	x	x
Methods											
Study design	4	Present key elements of study design early in the paper		x	x		x			x	x
Setting	5	Describe the setting, locations, and relevant dates, including periods of recruitment, exposure, follow-up, and data collection	x	x	x	x	x	x	x	x	x
Participants	6	(*a*) Give the eligibility criteria, and the sources and methods of case ascertainment and control selection. Give the rationale for the choice of cases and controls	x	x	x	x	x	x	x	x	x
		(*b*) For matched studies, give matching criteria and the number of controls per case	x			x	x		x		x
Variables	7	Clearly define all outcomes, exposures, predictors, potential confounders, and effect modifiers. Give diagnostic criteria, if applicable					x			x	
Data sources/ measurement	8	For each variable of interest, give sources of data and details of methods of assessment (measurement). Describe comparability of assessment methods if there is more than one group	x	x	x	x	x	x	x	x	x
Bias	9	Describe any efforts to address potential sources of bias	x				x		x		
Study size	10	Explain how the study size was arrived at	x	x	x	x	x		x	x	x
Quantitative variables	11	Explain how quantitative variables were handled in the analyses. If applicable, describe which groupings were chosen and why	x	x	x	x	x	x	x	x	x
Statistical methods	12	(*a*) Describe all statistical methods, including those used to control for confounding	x	x	x	x	x	x	x	x	x
		(*b*) Describe any methods used to examine subgroups and interactions	x	x	x	x	x	x	x	x	x
		(*c*) Explain how missing data were addressed									
		(*d*) If applicable, explain how matching of cases and controls was addressed	x			x					x
		(*e*) Describe any sensitivity analyses									
Results											
Participants	13	(a) Report numbers of individuals at each stage of study—eg numbers potentially eligible, examined for eligibility, confirmed eligible, included in the study, completing follow-up, and analyzed	x	x	x	x	x	x	x	x	x
		(b) Give reasons for non-participation at each stage	x	x					x	x	
		(c) Consider use of a flow diagram				x					
Descriptive data	14	(a) Give characteristics of study participants (e.g., demographic, clinical, social) and information on exposures and potential confounders					x		x	x	x
		(b) Indicate number of participants with missing data for each variable of interest									
Outcome data	15	Report numbers in each exposure category, or summary measures of exposure									
Main results	16	(*a*) Give unadjusted estimates and, if applicable, confounder-adjusted estimates and their precision (e.g., 95% confidence interval). Make clear which confounders were adjusted for and why they were included	x	x	x	x	x	x	x	x	x
		(*b*) Report category boundaries when continuous variables were categorized									
		(*c*) If relevant, consider translating estimates of relative risk into absolute risk for a meaningful time period									
Other analysis	17	Report other analyses done—e.g., analyses of subgroups and interactions, and sensitivity analyses									x
**Discussion**											
Key results	18	Summarize key results with reference to study objectives	x	x	x	x	x	x	x	x	x
Limitations	19	Discuss limitations of the study, taking into account sources of potential bias or imprecision. Discuss both direction and magnitude of any potential bias	x	x		x	x	x	x	x	x
Interpretation	20	Give a cautious overall interpretation of results considering objectives, limitations, multiplicity of analyses, results from similar studies, and other relevant evidence	x	x	x	x	x	x	x	x	x
Generalisability	21	Discuss the generalisability (external validity) of the study results	x	x	x	x	x	x	x	x	x
Other information											
Funding	22	Give the source of funding and the role of the funders for the present study and, if applicable, for the original study on which the present article is based	x			x			x		x

## Results

In the first place, emphasizing the formal aspects, of the nine articles analyzed, we find that six were published between 2019 and 2020. It is evident that three of them belong to Latin America within this period (Colombia, Uruguay and Mexico) and three to Europe (Portugal, France and Sweden). Two of them use Spanish and the remaining four use English.

According to the introduction section, all articles except one defined and explained the variables they were investigating. It was found that two articles evaluated psychological abilities, two psychological flexibility and two stress; while psychological wellbeing, emotional intelligence and cognitive abilities completed all findings. Regarding the programs used to measure these variables, it was found that four were developed by the authors themselves, while the other five were replicated programs. Likewise, only one article omitted the mention of research objectives. Besides, three studies presented specific objectives. Finally, it was found that in five of the nine articles the authors mentioned hypotheses.

Regarding the aspects found in the method, four opted for a quasi-experimental design, followed by two experimental studies, a case series study and a couple of articles that did not specify any design. However, only four studies were able to explain the design. Additionally, it was confirmed that all had a pre and post intervention evaluation. According to the participants, it was found that five articles had only men as a sample, two exclusively women, and two other studies participated both genders. Regarding age, it should be noted that only four articles showed their basic measures (range, mean, standard deviation). For their part, it was found that two articles selected practitioners of various sports, while another two chose football as the sports practice to be investigated. The remaining sports were Ice Hockey, Field Hockey, Volleyball, Rugby, and Artistic Gymnastics. Regarding the instruments, seven studies showed evidence of validity and reliability. Finally, in relation to the procedure, all the articles except one described or made a design of the sessions of the applied program.

Regarding the results section of these nine selected articles, it was found that four of them presented descriptive statistics, only one presented correlations between the investigated variables, and another five articles made comparisons. Additionally, six studies worked out the effect size, while the remaining ones were limited to comparing the results obtained from the pre and post-test.

Finally, referring to the discussion section, the five studies that presented hypotheses were able to confirm it and give a logical explanation. In addition, the results they obtained were analyzed and compared with other research in all articles. Finally, the nine selected studies concluded that the applied program had a positive impact on one or more of the psychological variables investigated (Tables 2, 3).

**Table 2:** Descriptive summary of the analyzed studies.

**N°**	**Título**	**País**	**Idioma**	**Año**	**Objetivos**	**Diseño**	**Variables**	**Programa**	**Deporte**	**Muestra**	**Edad**	**Conclusiones**
1	Acceptance and Commitment Training to Promote Psychological Flexibility in Ice Hockey Performance: A Controlled Group Feasibility Study.	Sweden	English	2020	The specific aim of this study is to investigate the feasibility and effect of an ACT training program for ice-hockey players	Experimental	Psychological flexibility	Acceptance and Commitment Training 4 sessions of 30–40 mins for 4 weeks	Ice Hockey	21 men EG: 13 CG: 8	R: – M: 26.29 SD: 5.14	The present study suggests that the application of a ACT program adapted to the ice hockey context is considered useful and meaningful for ice hockey players.
2	Programa de entrenamiento deportivo sobre variables cognitivas en deportistas de selección colombiana de gimnasia artística. Serie de casos	Colombia	Spanish	2020	The objective was to determine the effect of a training program (Mentality) on cognitive variables (time reaction, decision-making and the attention volume) in the athletes belonging to the Colombian men's artistic gymnastics team.	Case series study	Time reaction Decision-making Attention volume	Mentality Program 96 sessions of 30 mins for 6 months	Artistic gymnastics	8 men	R: – M: 21.6 SD: 1.06	With the application of the mentality program they were given psychological tools that allowed improvements in the skills under study, reflecting in better performance during the training units and competitions after the development of this research, confirming the principle that the skills.
3	Programa de entrenamiento en habilidades psicológicas en jugadoras de voleibol de alto rendimiento	Peru	Spanish	2012	The objective of this work is to present the results of the execution of the intervention program “sports psychological skills” carried out with the members of the Peruvian Women's Volleyball Team, minor category.	Not specify	Psychological skills	Sport Psychological skills 29 sessions of 45 mins for 4 months	Voleyball	15 women	R: 13–16 M: – SD: –	The psychological intervention program “Sports Psychological Skills” produced learning and modifications in the various skills and resources taught, as in confidence and in the sense of the practice of sport, which not only helped them to improve in the sporting aspect but also in the personal and academic sphere, as stated in their exit reports.
4	Mindfull Compassion Training on elite soccer: effects, roles, and associations on flow, psychological distress and thought suppression	Portugal	English	2019	Current study explored relations among self-compassion, mindfulness, psychological flexibility, and psychological distress (including anxiety), thought suppression and flow state, through the implementation of Mindfulness-Based Soccer Program (MBPSoccerP) for elite athletes.	Cuasi-experimental	Psychological flexibility Psychological distress Thought suppression Flow	Mindfull Compassion Training 9 sessions of 90 to 120 mins for 8 weeks	Soccer	57 men EG: 28 CG: 29	R: 18–30 M: 25.68 SD: 3.42	Results suggest that mindfulness, self-compassion and psychological flexibility development may be beneficial in cultivating positive sport experience and flow state and less psychological distress and thought suppression.
5	Evaluación e intervención psicológica en jugadoras de hockey sobre hierba femenino	Uruguay	Spanish	2020	The objective of the study is to identify the psychological profile of high-performance female hockey players through an assessment of psychological abilities. In addition, to observe if after the intervention there is an improvement in the psychological skills that intervene in the sports performance of field hockey players.	Cuasi-experimental	Psychological skills	Psychological Training 16 sessions of 60 to 80 mins for 4 months	Grass Hockey	10 women	R: 16–26 M: 21 SD: 3.71	After the cognitive behavioral psychological intervention, over 4 months and 16 sessions, with techniques and strategies such as self-characterization; the Stroop and Concentration Grid technique; communication skills and self-knowledge; behavioral self-records; observations, confrontations and interpretations; and relaxation and visualization techniques, better management of stress typical of competition and training is achieved, with reports of satisfaction with what has been learned and integrated.
6	Facilitating dual careers by improving resistance to chronic stress: effects of an intervention programme for elite student athletes	Germany	English	2018	The objective of this study was to evaluate the effectiveness of SRT-EA	Cuasi-experimental	Stress-resistance	Stress-resistance training for elite student athletes (SRT-EA) 10 sessions of 90 mins for 10 weeks	Varios	245 between men and women EG: 128 CG: 117	R: 13–20 M: 16.38 SD: 1.26	SRT-EA seems to be a suitable tool for broad and sustainable facilitation of individual resistance to chronic stress. It can be seen as an alternative to universal life-skills programmes for EA and a complement to interventions with a focus on acute stress and/or psychological skills in sports
7	Effect of Mindfulness on the Stress–Recovery Balance in Professional Soccer Players during the Competitive Season	Mexico	English	2020	This work examines the effect of six weeks of MSPE on the stress–recovery balance in professional soccer players during a competitive season, using RESTQ-76 Sport and HRV as psychometric and physiological evaluation methods.	Cuasi-experimental	Stress-recovery balance	Mindful Sports Performance Enhancement (MSPE) 6 sessions of 15 to 45 mins for 6 semanas	Soccer	42 men EG: 20 CG: 22	R: – M: 17.15 SD: 1.3	Six weeks of MSPE improves the stress–recovery balance, measured by the psychometric questionnaire RESTQ-Sport 76 in third-division professional soccer players during the competitive season, reduces stress, and increases recovery.
8	Percepción del bienestar y de la salud psicológica, y la eficacia de un programa de intervención en coaching en deportistas de rendimiento	Spain	Spanish	2016	The objectives of this work are to evaluate the wellbeing and psychological health, as well as to study the differences before and after the intervention of a coaching program on these variables in performance adult athletes.	Experimental	Psychological wellbeing Psychological health	Coaching Intervention Program Between 4 to 12 sessions (depends on each person) of 45 to 90 mins, between 1 to 3 months	Many	61 between men and women EG: 31 CG: 30	R: 18–40 M: 22.99 SD: 6.59	The EBP and GHQ-12 instruments have been shown to be useful and reliable for the evaluation of psychological wellbeing and general and emotional health in athletes; The practice of performance sports can positively affect the development of the wellbeing of athletes, although in turn it can negatively influence their psychological health; coaching can be effective as an intervention strategy in behavior modification and improvement of psychological health.
9	Emotional Intelligence (EI) Training Adapted to the International Preparation Constraints in Rugby: Influence of EI Trainer Status on EI Training Effectiveness	France	English	2019	The aim of this study was to investigate the effectiveness of an EI training program that is adjusted to the time restrictions of international rugby players and included within the training week preceding a competition such as regular conditioning sessions. technical or physical.	Not specify	Emotional intelligence	Emotional Intelligence (EI) Training Adapted to the International Preparation Constraints in Rugby 3 sessions of 60 mins for 3 days	Rugby	96 men EG 1: 23 EG 2: 24 EG 3: 22 CG: 23	R: 17 M: – SD: –	In the current study, the expert in sports psychology was able to improve the players' emotional regulation skills; the coach improved the players' ability to express their emotions, while the physiologist improved their skills to use the emotions of others. Therefore, our findings showed for the first time that the effects of EI training were influenced by the person who delivered the program.

**Table 3:** Summary of analysis of selected studies.

**N°**	**V**	**D**	**R**	**E**	**O**	**C**	**P**	**Ed**	**VC**	**Ds**	**Rd**	**Rr**	**Rc**	**T**	**Ci**	**Ip**
1	Yes	Yes	Yes	No	Yes	No	Yes	Yes	Yes	Yes	No	No	No	Yes	Yes	Yes
2	Yes	Yes	No	Yes	No	No	Yes	No	No	Yes	No	No	Yes	No	Yes	Yes
3	Yes	Yes	No	Yes	Yes	No	Yes	No	No	Yes	No	No	Yes	No	Yes	Yes
4	Yes	Yes	Yes	No	Yes	Yes	Yes	No	Yes	Yes	Yes	Yes	No	Yes	Yes	Yes
5	No	No	No	Yes	Yes	No	Yes	Yes	Yes	Yes	Yes	No	No	No	Yes	Yes
6	Yes	Yes	Yes	No	Yes	Yes	Yes	Yes	Yes	Yes	Yes	No	Yes	Yes	Yes	Yes
7	Yes	Yes	Yes	No	Yes	Yes	Yes	No	Yes	Yes	No	No	No	Yes	Yes	Yes
8	Yes	Yes	No	Yes	Yes	Yes	Yes	Yes	Yes	Yes	Yes	No	Yes	Yes	Yes	Yes
9	Yes	Yes	No	Yes	Yes	Yes	Yes	No	Yes	No	No	No	Yes	Yes	Yes	Yes

V, Variables in the title; D, Variable's definition; R, Replicated program; E, Own elaboration program; O, Mention of objectives in introduction; C, Hypothesis confirmed; P, Pre and post-test; Ed, Design explication; VC, Validity and Reliability; Ds, Sessions' description; Rd, Descriptive results; Rr, Correlational results; Rc, Comparative results; T, Effect size; Ci, Comparison between investigations Ip, Program positive program.

## Discussion

The purpose of this review was to synthesize and analyze the scientific studies between the period 2010-2020 that have evaluated the effects of psychological interventions on the performance of professional athletes who perform in highly competitive settings. In particular, nine studies were selected from a total of 632 identified in the corresponding databases. This number found within a large universe of studies makes us reflect on intervention designs in scientific research. Based on the filters developed and the literature found, it is evident that, in sports psychology, non-experimental studies predominate over those that study the effects of programs (quasi-experimental or experimental). Regarding this, a review that analyzed the current state of research on sports psychology in Spanish between 2010 and 2016, found that the trend was non-experimental designs with a total of 60% of selected articles; although between quasi-experimental and experimental investigations accounted for 24% of the total (Calderón and Lesmes, 2016). It leads us to reflect on whether the scope of the applied psychological intervention programs.

A second important finding was regarding the year of publication of the articles, because only in the last couple of years (2019 and 2020) more studies were found than in the previous nine (2010–2018). This could represent the possible lack of psychological intervention work in the last decade; or we could deduce that there are not many records of this type of investigation o there are great difficulties in systematizing and drafting such intervention. In this way, it would not necessarily mean that interventions of this nature have not been carried out in the sports field, but rather that they are not reflected in high-impact magazines; and only in recent years could they be published by these media. The dissemination of research in sports psychology is very important because it provides valuable information and knowledge based on evidence that allows solving multiple relevant practical questions to optimize the sports performance of athletes (Moran and Toner, 2017). Additionally, this finding evidences that there is a growth in the publication of sports psychological interventions in high impact magazines, improving the visibility of the results related to the improvement of the performance of elite athletes. On the other hand, it is important to emphasize that, according to the search period of the Ursino and Barrios (2019) review carried out from 2008 to 2018, it was found that the highest concentration of studies that associated sports performance with psychological variables was between 2015 and 2018, repeating the pattern that there is more research in the last years of the search period, although not exclusively in experimental or quasi-experimental designs, but in empirical studies in general.

As a third finding, the most investigated variables in these nine articles were: psychological abilities, psychological flexibility, and stress. However, it is relevant to consider that, in one of the studies, psychological flexibility was not the only variable investigated, but was shared by others (psychological distress, thought suppression and Flow). In addition, with respect to stress, it was evaluated in different dimensions, because one article emphasized resistance to stress and the other on the stress-recovery balance. In this way, it is evident that the evaluation of psychological abilities had a greater role among the others. It is likely that this is due to the fact that psychological abilities group several constructs that have been scientifically proven over the years that directly intervene in sports performance, such as motivation, attention, coping, attitude, anxiety, imaginative visual control, among other (Getz and McConnell, 2014; León et al., 2014; Ramos-Cabal et al., 2018). Furthermore, the importance of psychological skills in sports performance has been demonstrated by many researchers (MacNamara et al., 2010; López-Gullón et al., 2011; Weinberg and Gould, 2014; Abdullah et al., 2016; Ramírez-Muñoz and Prieto-Andreu, 2021). Not only psychological skills may provide you a general psychological profile of high-performance athletes, but also to know which the variables are to work on according to the extent to which they influence their competitive performance (Loehr, 1986; Raimundi et al., 2016).

Regarding the programs implemented in the different research, the use of already established programs predominates (55%), compared to those elaborated for the purposes of the studies; understanding that there are highly recognized psychological techniques used in the construction of programs, such as Mindfulness.

This type of psychological interventions within the sports field have been described as emerging third generation therapies with high levels of efficacy. Demonstrating improvements in athletes who favored different states of anxiety and concentration (Hoja and Jansen, 2019). A similarity found in two replicated programs was the use of this technique to address psychological skills such as flexibility, thought suppression, flow, and stress. The continuous investigation of this psychological technique is what has allowed us to know and verify its multiple benefits. Therefore, intervening with this type of (replicated) programs that already have a long history and scientific support, allows to obtain greater security with respect to the development of the psychological skills investigated; unlike what it could mean to implement a program with its own design that does not have the necessary evidence to know whether it can generate a positive impact. However, it is known that one of the main objectives of the research is not only to clarify and expand the knowledge that already exists, but also to generate new ones (Ato et al., 2013). This would be achieved through the implementation of self-made programs because it would be creating a new opportunity to investigate other options. In turn, improvements have also been observed with more classical second generation treatments or strategies, based on cognitive-behavioral therapies (Olmedilla et al., 2010). Therefore, the remaining 45% of the selected interventions could respond to designs developed by the researchers but using techniques that respond to theoretical models already consolidated in different contexts over time, allowing them to be adapted to the sports environment (Reyes-Bossio et al., 2012) y a otros niveles deportivos (Trujillo-Torrealva and Reyes-Bossio, 2019; Cárcamo-La Torre and Reyes-Bossio, 2022).

Another relevant finding was the great variety in terms of the age range of the athletes considering all the studies, since the youngest was 13 years old and the oldest 40 years old. This finding can have several explanations. The first is defined according to the category of athletes, because they may have selected juniors from the national teams they represent, such as a volleyball sub-17. There are other athletes who, instead of representing a youth category, play in professional teams or clubs of the first/second division of their national leagues, in which it is known that there is a greater variety in age groups. Also, it is important to consider the sport practiced, since there is a recognized age and average start and end of a career in each one (López de Subijana and Equiza, 2018). As was previously known, of the nine studies selected, six different sports were found, which is why in some the age of the athletes may be dispersed. Finally, it should be noted that, in addition to the six sports found, two studies evaluated athletes from various sports, which could also explain the variance in age. Additionally, regarding the sex of the athletes, it was found that more than half of the studies intervened only with men, coinciding with the systematic review by Brown and Fletcher (2017). However, 44% of the articles had the presence of women in their samples. Although these findings appear to be encouraging in the field of scientific research, there is still a large gap in the resources allocated to sportsmen and women, especially financial ones, generating inequity (López, 2017).

Following the line of sports, soccer was the sport with the greatest presence in the different studies. Although the magnitude and beauty of this sport around the world is known, from a total of nine studies, five other sports disciplines were found. This may reflect the increasing awareness, research, and psychological intervention in various sports. Added to this, it is worth mentioning that all the sports presented were collective, except for artistic gymnastics, which has a mixed modality. Within the search process of the present review, several psychological interventions were found on individual sports, such as table tennis, sailing, taekwondo, among others; but they were logically investigated under a single case design, falling outside of our inclusion criteria. However, this type of research, which seeks to understand the behavior of an individual in response to an intervention program, is progressively increasing in the psychology of sport and exercise (Kratochwill and Levin, 2010; Moran and Toner, 2017). For more detail, you can examine the review by Barker et al. (2011), who investigated the nature and applications of single case designs in that discipline.

A sixth finding was that three studies chose to implement their psychological training program to their entire sample, understanding that they only had one intervention group. Therefore, it is logical that they have chosen to make comparisons between the evaluations applied before and after the program to determine their impact. In contrast, the other six studies divided their sample into an experimental and control group, applying their program only and obviously to the first of the groups. Unlike the other three studies, these six had and made effective the possibility of using the effect size statistic, not only to assess whether there was improvement in the psychological variables evaluated after the intervention, but also in what extent. This means a very relevant result considering that it allows to know how much the findings can be adjusted to reality (Castillo and Bravo, 2015).

Finally, in order to evaluate the psychological work resulting from these interventions, it is important to focus on the improvement of the psychological functioning of athletes, assessing their adaptive progress, their relationship with performance and their psychological wellbeing (Olmedilla and Domínguez-Igual, 2016; Kosendiak and Ptak, 2017). All articles concluded that their psychological training programs had a positive impact on high-performance athletes, regardless of the type of program, intervention strategy or technique used, as well as without discriminating in the distribution or characterization of each sample, coinciding with the findings of the Brown and Fletcher (2017) review. Perhaps this level of improvement as a consequence of the different interventions selected may be due to the level of satisfaction with the psychological training by both the athletes and the technical staff, as they were able to find time to reflect, share, enjoy and learn techniques such as relaxation, mindfulness and visualization. We could think that generating spaces for exchange and experimentation have effects on the improvement of skills and sense of wellbeing. Similar results were found with Polish Olympic athletes (Kosendiak and Ptak, 2017) alluding to their enjoyment of the choice of relaxation and visualization techniques, within a psychological intervention program. However, it is important to clarify, as mentioned above, that some studies evaluated several psychological variables at the same time, so this finding is focused on the overall effectiveness of the program, but not necessarily on the significant improvement of all the psychological constructs addressed. Therefore, the authors culminated their studies with the request and recommendation to continue researching on these topics in order to clarify the panorama and obtain new discoveries that contribute to the growth of sport psychology. At the end of the day, it is the publications of this type of research that allow society to have more knowledge and resources at its disposal for its interventions.

### Limitations

A first limitation of the study was to discriminate and define the characteristics of a high-performance athlete over one who is not. This is because several aspects are involved, such as age, category, representativeness in a national team and participation in a professional team. In this way, it was difficult to select the studies that presented the characteristics of the required sample.

Added to this, in the beginning it had been planned to select all kinds of research that would demonstrate the effects of psychological training, including qualitative ones and case studies. However, in order to obtain greater uniformity in the results and analysis, it was decided to select only the quantitative studies. It happens that qualitative studies have other elements that could be discussed and analyzed. Such information, together with what was found in quantitative research, was going to mean more extension, diseños y posibles confusiones metodológicas.

Another limitation is found in the predominance of non-experimental descriptive cross-sectional designs. There are even studies that make mental or psychological training programs available, but a scientific evaluation of the impact generated on athletes is not observed. This is connected with the lack of rigor in the results supported by scientific evidence, understanding that there may be a large number of sports psychological interventions, but that they do not necessarily go through the rigorous process of scientific research, and this causes that ultimately there is no support for a positive impact.

### Practical implications

The present study will clarify and have relevant information available on the impact of psychological interventions on sports performance. In this way, it can contribute to different sports institutions having knowledge and providing themselves with various resources and activities that favor the development of psychological skills related to improving their performance. They will have a wide range of effective interventions to apply with their own athletes according to the required needs.

### Recommendations

In the first place, based on the objective of promoting the continuous obtaining of new discoveries based on scientific evidence, especially in sports psychology, we recommend continuing research on this discipline in the process of consolidation. The results and analysis that we do cannot be taken as absolute truths because our investigations are framed in a specific sample and context, so it would be a mistake to generalize to large magnitudes. Therefore, to approximate reality, there needs to be a series of studies to support it, and this will only be achieved if there is continuous scientific research.

It is recommended that the inclusion criteria be fairly clear and free of interpretation, because given the large amount of research to be analyzed, some may go unnoticed due to a doubtful inclusion criterion. Furthermore, it makes it difficult for the funnel process, shown in the PRISMA diagram, to be fast and effective.

Finally, it is suggested to analyze and investigate the challenges framed in sports psychology after the Covid-19 pandemic. Because possibly the needs and demands of athletes have mutated and now the psychological variables to work on could be different from what they were a couple of years ago. Athletes and society in general are in constant readjustment processes due to the new competition conditions and sports psychology professionals must consider them in their new interventions both face-to-face and virtual, which has come to stay and be enhanced with technology.

## Conclusions

There has been a recent and accelerated growth in the research of interventions in sports psychology, which shows that both professionals in this discipline and sports institutions are opting for their work to have a greater reach in society and form part of the required scientific evidence to be replicated with sustenance. Additionally, it is relevant to evaluate the psychological abilities in high-performance athletes, because by grouping a series of (proven) variables that influence their performance, you obtain a psychological profile of the athlete that will allow you to know what aspects could be worked on to have the maximum potential. Finally, from all the information collected and analyzed, it is concluded that interventions on psychological variables have a positive impact on the performance of highly competitive athletes.

## Data availability statement

The raw data supporting the conclusions of this article will be made available by the authors, without undue reservation.

## Author contributions

MR-B conceived the original idea of the study. MR-B, GV-S, MD-C, PB-A, and LC-A selected the references and contributed to data selecting and processing. MR-B, SC-B, and MV analyzed and presented the data. MR-B, TC-R, VT-V, CC-L, and RB wrote and organized the manuscript. All authors have read and agreed to the published version of the manuscript.
